# Parenting stress and Chinese preschoolers’ approaches to learning: a moderated mediation model of authoritative parenting and household residency

**DOI:** 10.3389/fpsyg.2023.1216683

**Published:** 2023-08-07

**Authors:** Jinghui Zhao, Yizhen Fan, Ziqin Liu, Chaopai Lin, Limin Zhang

**Affiliations:** ^1^Department of Early Childhood Education, School of Education, Guangzhou University, Guangzhou, China; ^2^Department of Early Childhood Education, School of Education, Central China Normal University, Wuhan, China

**Keywords:** parenting stress, Chinese preschoolers’ approaches to learning, authoritative parenting, household residency, parenting style

## Abstract

According to the family stress model, this study examined the relationship between parenting stress and preschoolers’ approaches to learning (ATL) in China, as well as the mediating effect of authoritative parenting and the moderating effect of household residency (migrant and native). A survey of 5,047 preschoolers’ parents (2,186 natives and 2,861 migrants) supports the proposed moderated mediation model. The results showed that after controlling for gender and age, parenting stress affected preschoolers’ development of ATL negatively. Authoritative parenting mediates the relationship between parenting stress and preschoolers’ ATL. Further, household residency moderated the relationship between authoritative parenting and preschoolers’ ATL. The findings of this study suggest that high levels of parenting stress are detrimental to the development of preschoolers’ ATL. And parents with low parenting stress are more likely to adopt authoritative parenting, which in turn fosters preschoolers’ ATL. In addition, native families’ authoritative parenting style are more conducive to fostering preschoolers’ ATL than migrant families. Finally, this study contributes to previous research by examining the mechanisms of parenting stress on preschoolers’ ATL and provides support for the extension of the family stress model. Importantly, it also informs efforts to improve ATL among preschoolers in Chinese migrant and native families.

## Introduction

Over the past years, approaches to learning (ATL) has attracted considerable attention and become a common demand in early childhood education in developed countries (Moffett et al., 2023; Stephens et al., 2023). ATL refers to children’s attitudes, habits, learning styles, and behaviors as they engage in educational activities and achieve goals (Kagan et al., 1995; McDermott et al., 2016). Specifically, they describe how children learn rather than what children have learned. Up to now, many studies have explored the factors of ATL. For example, in the Early Childhood Longitudinal Study-Kindergarten Class of 1998–99 (ECLS-K), ATL includes factors such as organization, attentiveness, learning independence, persistence, flexibility, responsibility, and creativity (U.S. Department of Education and National Center for Education Statistics, 2002). In addition, Scott-Little and colleagues’ research suggested that ATL’s factors include curiosity, initiative, persistence, attentiveness, etc. (Scott-Little et al., 2006). Moreover, it has been confirmed by most researchers that ATL involves critical factors such as curiosity, initiative, persistence, and creativity. There is currently a substantial body of longitudinal studies suggests that ATL is essential to children’s later academic achievement, including reading, vocabulary, language, mathematics, science, etc. (Li-Grining et al., 2010; McClelland et al., 2013; McDermott et al., 2014; Bustamante et al., 2017; Sung and Wickrama, 2018). Interestingly, prior research has proven relationships between ATL and other aspects of children’s development, such as peer relationships, social competence, and executive function, etc. (Coolahan et al., 2000; Razza et al., 2015; Sung and Wickrama, 2018; Moffett et al., 2023). Furthermore, some previous studies have suggested that early ATL may serve as a source of resilience for children who have been exposed to risk factors (Luthar et al., 2000; Li-Grining et al., 2010). Hence, it is crucial to recognize and foster ATL, especially as they emerge in the key preschool years (McDermott et al., 2002).

There is no doubt that various family factors influence children’s development in different ways. According to the extension of the family stress model, parents’ psychological distress can influence a child’s outcome via disrupted parenting (Conger et al., 2002; Masarik and Conger, 2017). In spite of the fact that the family stress model has historically focused on economic stressors, it can also be applied to other stressor variables including parenting stress (Masarik and Conger, 2017; Marcal, 2022). Further, previous studies have shown that parenting stress can negatively affect the ATL of children (Chazan-Cohen et al., 2009; Smith-Adcock et al., 2019). Moreover, a longitudinal study has reported a link between lessening parental stress and better ATL for the first 5 years of a child’s life (Chazan-Cohen et al., 2009).

However, the influence of parenting stress on preschoolers’ ATL has not been fully explored. Preschoolers’ ATL may be affected directly or indirectly by parenting stress. Directly, children are sensitive to their parents’ emotional states, so when their parents experience high levels of stress, they may also experience tension, stress, or anxiety. This negative emotion can hinder their motivation and willingness to learn (Chazan-Cohen et al., 2009; Herba et al., 2016; Smith-Adcock et al., 2019). Indirectly, parenting stress may reduce parent-school involvement and the quality of parent–child interaction, lead to maladaptive parenting behaviors such as punishment and harsh response, and especially lead parents to be more authoritarian or permissive toward their children, which in turn affects preschoolers’ ATL (Fonseca et al., 2020). In addition, little research has been done into a moderated mediation model that incorporates ATL, parenting stress, and other family factors. Guided by previous research, in the current study, we draw from the family stress model to build a comprehensive model that links parenting stress to authoritative parenting style, preschoolers’ ATL, and household residency (Chinese migrant families and native families). As well as further investigate how parenting stress affects preschoolers’ ATL. In addition, we further reveal the potential mediating roles of the authoritative parenting style in the association between parenting stress and preschoolers’ ATL. Further, we also examine whether household residency may serve as a moderator of the relationship between authoritative parenting and preschoolers’ ATL. Specifically, we examine which household residency in the cities may enhance the positive effect of authoritative parenting on preschoolers’ ATL. Lastly, our study provides significant recommendations regarding preschoolers’ ATL in Chinese migrant and native families.

## Literature review and hypotheses

### Parenting stress and preschoolers’ approaches to learning

The concept of parenting stress is generally understood to be a negative psychological reaction that occurs when parents are not able to meet their parenting needs and concerns about their role as parents (Deater-Deckard, 1998). Our study focuses on parenting stress in parents of preschoolers because this developmental period poses a number of challenges for them (Harmeyer et al., 2016). Although many parents experience parenting stress, multiple studies show that high parenting stress can lead to a range of child development problems (Fang et al., 2022). When parents are experiencing high levels of parenting stress, they may become less responsive, inconsistent, or harsh to the needs of their children (Crnic and Low, 2002). These negative parent–child interactions can result in a variety of children’s developmental problems, including difficulties with adjustment, problem behaviors, language delay, sleep problems, and negative emotions (Deater-Deckard, 1998; Baker et al., 2003; Horwitz et al., 2003; Martin et al., 2019). Additionally, children raised by parents with high parenting stress may have lower cognitive abilities and poor academic performance due to fewer positive learning interactions and stimulations in the family daily life (Tachibana et al., 2012). Furthermore, these negative effects caused by parenting stress during early childhood may influence preschoolers’ future development (Neece et al., 2012). According to a recent study, parents’ low perceived social support and negative emotions negatively affect their children’s ATL (Yan et al., 2022). Moreover, low perceived social support is generally associated with parenting stress (Huang et al., 2014). Further, parenting stress, which could cause more frequent negative emotions in parents, may adversely affect the development of preschoolers’ ATL (Fonseca et al., 2020). Hence, this study proposes the following hypotheses based on the information provided above:

*H1*: Parenting stress negatively influences preschoolers’ ATL.

### Authoritative parenting as a mediator

When assessing parenting stress’ impact on preschoolers’ ATL, it is imperative to consider how parents respond to this stressful situation to reduce the negative impact on preschoolers. Multiple research suggests that parental parenting style is crucial to a child’s development, particularly during the preschool years (Tan et al., 2012; Hosokawa and Katsura, 2019). Additionally, a large number of studies suggest that parental stress influences children’s development through its effects on parenting (Crnic et al., 2005; Tan et al., 2012). Interestingly, a substantial amount of literature has examined that parents with lower parenting stress are more likely to adopt authoritative parenting (Park and Walton-Moss, 2012; Gouveia et al., 2016; Fonseca et al., 2020). Most studies have described authoritative parenting as more likely to include a higher degree of restriction, responsiveness, warmth, developmental appropriateness, and support than other parenting styles (Baumrind, 1971; Robinson et al., 1995; Park and Walton-Moss, 2012; Gouveia et al., 2016). Authoritative parents pay close attention to their children’s feelings and communicate with them frequently. In addition to granting their children reasonable autonomy, authoritative parents also teach them how to make wise decisions (Pinquart, 2017). Moreover, ample research has demonstrated that authoritative parenting is a type of positive parenting that fosters children’s development, such as prosocial engagement, cooperation, social competence, peer acceptance, school achievement, etc. (Crnic and Low, 2002; Cheah et al., 2009). Furthermore, a recent Chinese study found that authoritative parenting can positively predict preschoolers’ ATL (Xia, 2023). In addition, a systematic review indicates that parental supportive and warm behaviors can act as protective factors to increase preschoolers’ appropriate behaviors and decrease problematic behaviors when parents face high levels of economic stress (Masarik and Conger, 2017). Based on the above, authoritative parenting may serve as a protective factor buffering the negative impact of parenting stress on preschoolers’ ATL development. However, to date, only a few studies have explored the relationship between authoritative parenting style in terms of parenting stress and preschoolers’ ATL. As such, this study proposes the following hypothesis:

*H2*: Authoritative parenting mediates the relationship between parenting stress and preschoolers’ ATL.

### Household residency as a moderator

In recent years, as the Chinese economy has developed rapidly and urbanization has accelerated, the number of Chinese migrants from rural areas to urban areas has increased (Yang, 2013). Although migrants are generally defined as having crossed national borders, it is necessary to examine the impact of rural–urban migration on the development of preschoolers due to the enormous urban–rural divide in China (Yan et al., 2022). In this study, a migrant family is one in which both parents have rural residency and have lived in the city for more than half a year without obtaining a long-term residence permit. Moreover, there is considerable literature arguing that migrant and native parents have different parenting beliefs and behaviors, especially when it comes to their children’s education (Fibbi and Truong, 2015). Specifically, migrants may differ from native parents in terms of parenting self-efficacy, attitudes toward their children’s education, expectations, resources, involvement in their children’s daily activities, provision of cognitive stimulation, attitudes toward their children’s abilities and performance, etc. (Barglowski, 2019). Further, it has been demonstrated in previous studies that these differences can impact children’s development, particularly their ATL development (Padilla and Ryan, 2020). Accordingly, parenting outcomes and preschoolers’ ATL development may be significantly different in these two household residencies. Furthermore, according to Mistry and colleagues’ study, household residency can also serve as a moderator (Mistry et al., 2008). Thus, the present study predicts household residency might moderate the association between authoritative parenting and preschoolers’ ATL and proposes the following hypothesis:

*H3*: Household residency moderate the mediating effects of authoritative parenting style in the relationship between authoritative parenting and preschoolers’ ATL. Specifically, we propose that native parents’ authoritative parenting might be more effective than migrant parents at fostering preschoolers’ ATL.

### Present research

**Figure 1:**
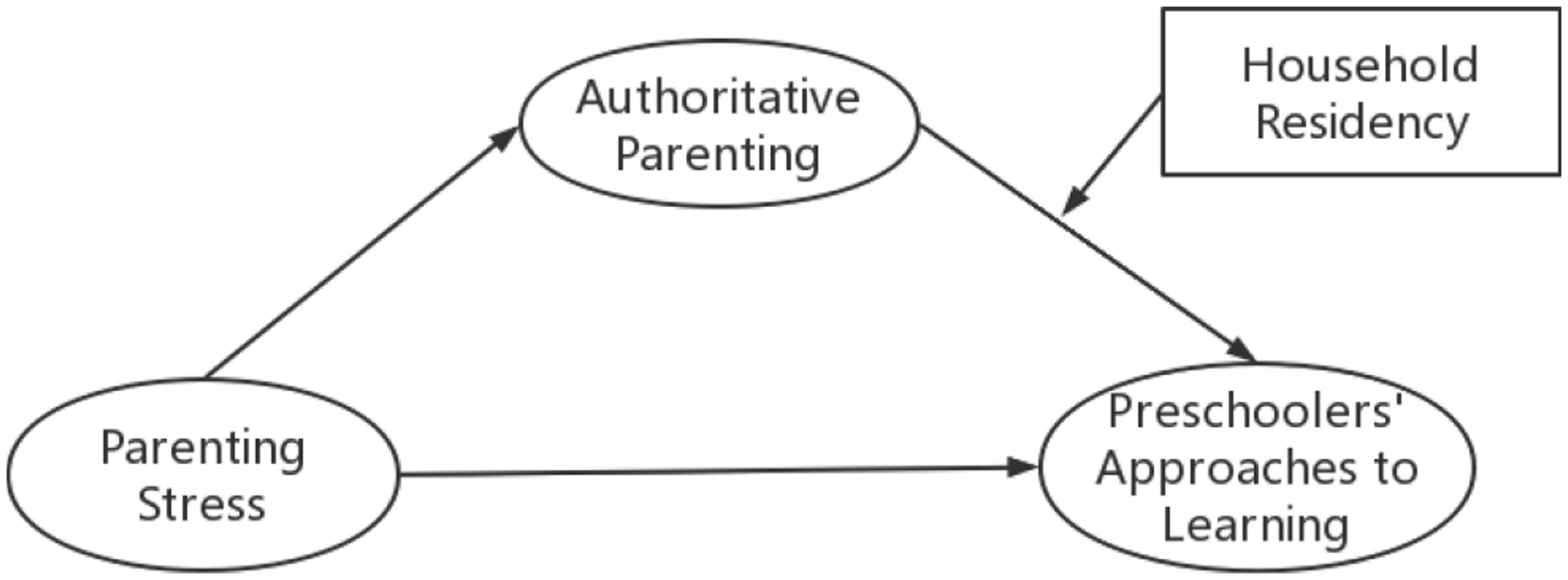
Hypothetical model.

The current study proposes a moderated mediation model (see Figure 1) on the basis of the theory of the family stress model. This study aimed to examine the relationship between parenting stress and preschoolers’ ATL. Furthermore, we also investigated how authoritative parenting plays a mediating role in parenting stress’ effect on preschoolers’ ATL. In this vein, how the influence of authoritative parenting on preschoolers’ ATL may differ based on household residency (migrant or native) was also explored.

## Materials and methods

### Participants

Using the convenience sampling method, this study selected parents from preschools that comprised both migrant and native preschoolers in Guangzhou, Foshan, and Shenzhen cities in southern China. We distributed 5,400 questionnaires and collected 5,047 valid responses, resulting in an effective response rate of 93.46%. The valid subjects included 2,186 native parents and 2,861 migrants. We obtained informed consent from all participants, and the Guangzhou University School of Education and related research ethics committee evaluated and approved the research. Specific demographic information is shown in Table 1.

**Table 1:** Demographic characteristics of participants (*N* = 5,047).

Statistical variables	Group	Frequency and percentage (%)	
		Native families	Migrant families
Number of participants	—	2,186 (43.31%)	2,861 (56.69%)
Gender of child	Boys	1,129 (51.65%)	1,532 (53.55%)
	Girls	1,057 (48.35%)	1,329 (46.45%)
Age range of child	3–4 years old	804 (36.78%)	708 (24.75%)
	4–5 years old	806 (36.87%)	998 (34.88%)
	5–6 years old	576 (26.35%)	1,155 (40.37%)
Parents’ educational level	High school or below	734 (33.58%)	1957 (68.41%)
	College graduates or above	1,452 (66.42%)	904 (31.59%)
Parents’ occupation	Manual workers/Unemployed	483 (22.10%)	1,445 (50.51%)
	Technical worker	54 (2.47%)	112 (3.90%)
	Self-employed/Freelancers	759 (34.72%)	712 (24.89%)
	Managers/Technicians	757 (34.63%)	508 (17.76%)
	Professionals/Executives	133 (6.08%)	84 (2.94%)
Household income (Annual)	Under 30,000 CNY (Under 4,138.79 USD)	197 (9.00%)	314 (10.98%)
	30,000–50,000 CNY (4,138.79–6,879.98 USD)	205 (9.38%)	414 (14.47%)
	50,000–100,000 CNY (6,879.98–13,795.96 USD)	433 (19.81%)	748 (26.14%)
	100,000–150,000 CNY (13,795.96-20,693.93 USD)	403 (18.44%)	574 (20.06%)
	150,000–200,000 CNY (20,693.93–27,591.91 USD)	316 (14.46%)	365 (12.76%)
	Over 200,000 CNY (Over 27,591.91 USD)	632 (28.91%)	446 (15.59%)
Residential environment	Suburb	281 (12.85%)	321 (11.22%)
	Urban village	428 (19.58%)	1,120 (39.15%)
	Older neighborhoods	482 (22.05%)	880 (30.76%)
	Apartment complex	995 (45.52%)	540 (18.87%)
Family socioeconomic status (SES)	Low family SES	584 (26.72%)	1,186 (41.45%)
	Middle family SES	645 (29.51%)	889 (31.07%)
	High family SES	957 (43.78%)	786 (27.47%)

1 USD = 7.2471 CNY (Exchange rate on July 06, 2023).

### Measures

#### Parenting stress

Parenting stress was assessed using the Chinese version of the Parenting Stress Index – Short Form (PSI-SF) (Abidin, 1995). Developed from the original Parenting Stress Index (PSI), the PSI-SF is a 36-item version of the original 120-item PSI (Abidin, 1983). The PSI-SF identifies three central factors of parenting stress: parental distress, parent–child dysfunctional interaction, and difficult child. Specifically, it is a self-report questionnaire that measures the stress experienced by parents with children aged 1 month to 12 years old. Since the PSI-SF has been used by multiple nonclinical and clinical groups as well as nationally representative samples, it has demonstrated excellent validity (Holly et al., 2019). Further, it was also confirmed that the Chinese version of the PSI-SF possessed good reliability and validity. Parents were asked to read the statement and answer on a 5-point Likert scale (1 is “strongly disagree” and 5 is “strongly agree”). A higher score indicates a higher level of parenting stress. In the current sample, the Cronbach’s α of the total PSI-SF was 0.889.

#### Authoritative parenting

Authoritative parenting was measured using one subscale of the Chinese version of the Parenting Styles and Dimensions Questionnaire (PSDQ) (Robinson et al., 1995). There are four dimensions of authoritative parenting style, which are assessed by 27 items, including “Reasoning/Induction” (7 items), “Good-natured/Easy Going” (4 items), “Warmth and Involvement” (11 items), and “Democratic Participation” (5 items). A five-point Likert scale was used to assess parents’ response to each item (where 1 means “never” and 5 means “always”). A higher score indicates that the parent uses authoritative parenting more frequently. It has been shown that the Chinese version has excellent internal consistency. In this study, Cronbach’s alpha of the Authoritative Parenting scale was 0.94.

#### Approaches to learning

A commonly used tool for measuring preschoolers’ ATL is the Preschool Learning Behavior Scale (PLBS) (McDermott et al., 2002). The PLBS is a 29-item scale composed of three reliable dimensions, Attention/Persistence (e.g., “Cannot settle into an activity”), Competence Motivation (e.g., “Tears when faced with difficulty”), and Attitude Toward Learning (e.g., “Unwilling to accept needed help”). In our study, we used the Chinese version of the PLBS, which showed good internal consistency in the previous study (Hu et al., 2017). It is a three-point Likert scale with three response options: 1 (does not apply), 2 (sometimes applies), and 3 (most often applies). A higher score on the scale suggests better ATL performance. In the current study, Cronbach’s α of the total PLBS was 0.836.

#### Demographic covariates

The parents reported their child’s age (1 = 3–4 years old, 2 = 4–5 years old, 3 = 5–6 years old), and their gender (0 = boy, 1 = girl). Both the child’s age and gender were included as covariates.

### Statistical analysis

In this study, data were analyzed using SPSS24.0, which included reliability analysis, common method bias test, descriptive statistics, and correlation analysis. We used Mplus8.3 to test structural equation models. And we tested the mediation model and the moderated mediation model using Bootstrap and multi-group comparison. Model parameters were estimated using the maximum likelihood method (ML). To assess the goodness of the model fit, we selected the following fit indices: the Tucker-Lewis index (TLI), the Comparative Fit Index (CFI), the Root Mean Square Error of Approximation (RMSEA), and the Standardized Root Mean Square Residual (SRMR). Since this study had a large sample size, the chi-square values were not used as a reference for the model fit. The cutoff values of CFI and TLI ≥ 0.9, SRMR and RMSEA ≤0.08 were adopted as the criteria for a good fit in the current study. And *p* value (*p*) < 0.05 was regarded as statistically significant (Kline, 2005).

To test whether household residency (native/migrant) has a moderating effect in the latter half of the mediation model, we used multi-group comparison to test the differences between groups. First, we examined the tested model between the two groups. We tested the following invariance: (1) Configural Invariance, that is, the attribution of indicators is equal among different groups; (2) Metric Invariance, that is, the loading of the indicator on the latent variable is equal between different groups; (3) Scalar Invariance, which means that the intercept of indicators is equal between different groups. In general, if the fit indexes (CFI, TLI, and RMSEA) do not change by more than 0.01, the model has not significantly changed (Meade et al., 2008). Following the testing of measurement invariance of the tested models, pathways between variables were added to the models. To test whether the pathways between authoritative parenting and preschoolers’ ATL are different among two family types, this study let these pathways be freely estimated among the two groups and constrained the invariance of other pathways. To determine whether the difference is significant, the Wald Test was used to compare the difference between the two pathways. If the Wald Test result has a significance *p*-value of less than 0.05, it suggests that the paths are statistically different across groups.

## Results

### Common method bias

Although this study uses a more mature measurement tool and emphasizes the confidentiality of personal information in data collection, according to previous studies on common method deviations, a Harman one-way test is required to confirm common method deviations statistically after data collection (Podsakoff et al., 2003). It was found that there were 13 factors with feature values greater than one. The variance explained by the first principal factor was 21.54%, which was less than the critical criterion of 40%. Thus, there is no common method bias in this study.

### Description statistics and correlation matrix

Table 2 presents the descriptive statistics (standard deviations and means), and correlations for the main study variables. Based on the data analysis results, parenting stress was negatively correlated with preschoolers’ ATL (*r* = −0.45, *p* < 0.01). In addition, parenting stress was also negatively associated with authoritative parenting (*r* = −0.37, *p* < 0.01). Further, authoritative parenting was positively associated with preschoolers’ ATL (*r* = 0.35, *p* < 0.01). Moreover, household residency was significantly associated with preschoolers’ ATL, parenting stress, and authoritative parenting (*r* = −0.09, *p* < 0.01; *r* = 0.16, *p* < 0.01；*r* = −0.09, *p* < 0.01, respectively).

**Table 2:** Descriptive statistics and correlation matrix for each variable (*N* = 5,047).

	1	2	3	4	5	6
1. Gender	—					
2. Age	−0.01	—				
3. Approaches to learning	0.03**	0.03**	—			
4. Parenting stress	0.00	0.05**	−0.45**	—		
5. Authoritative parenting	0.01	−0.04**	0.35**	−0.37**	—	
6. Household residency	−0.02	0.16**	−0.09**	0.16**	−0.09**	—
M	—	2.04	2.29	2.62	3.63	—
SD	—	0.80	0.24	0.43	0.58	—

**p < 0.01.

### Testing for the mediating role of authoritative parenting

This study tested the mediating effect of authoritative parenting based on the test procedure for mediation analysis of structural equations. Further, we estimated confidence intervals for each coefficient by the Bias-Corrected Bootstrap method (Bootstrap = 5,000), with 95% confidence intervals that do not contain zero indicating statistical significance (Shrout and Bolger, 2002). First, a simple regression model of latent variables was established to test whether parenting stress directly predicted preschoolers’ ATL. The results showed that the model fitted well with RMSEA = 0.05, CFI = 0.99, TLI = 0.98, and SRMR = 0.02. It was found that parenting stress negatively and significantly predicted preschoolers’ ATL after controlling for preschoolers’ gender and age (*b* = −0.30, *p* < 0.001). And the amount of preschoolers’ ATL explained by parenting stress was 37.3%. Hence, hypothesis 1 was supported.

**Figure 2:**
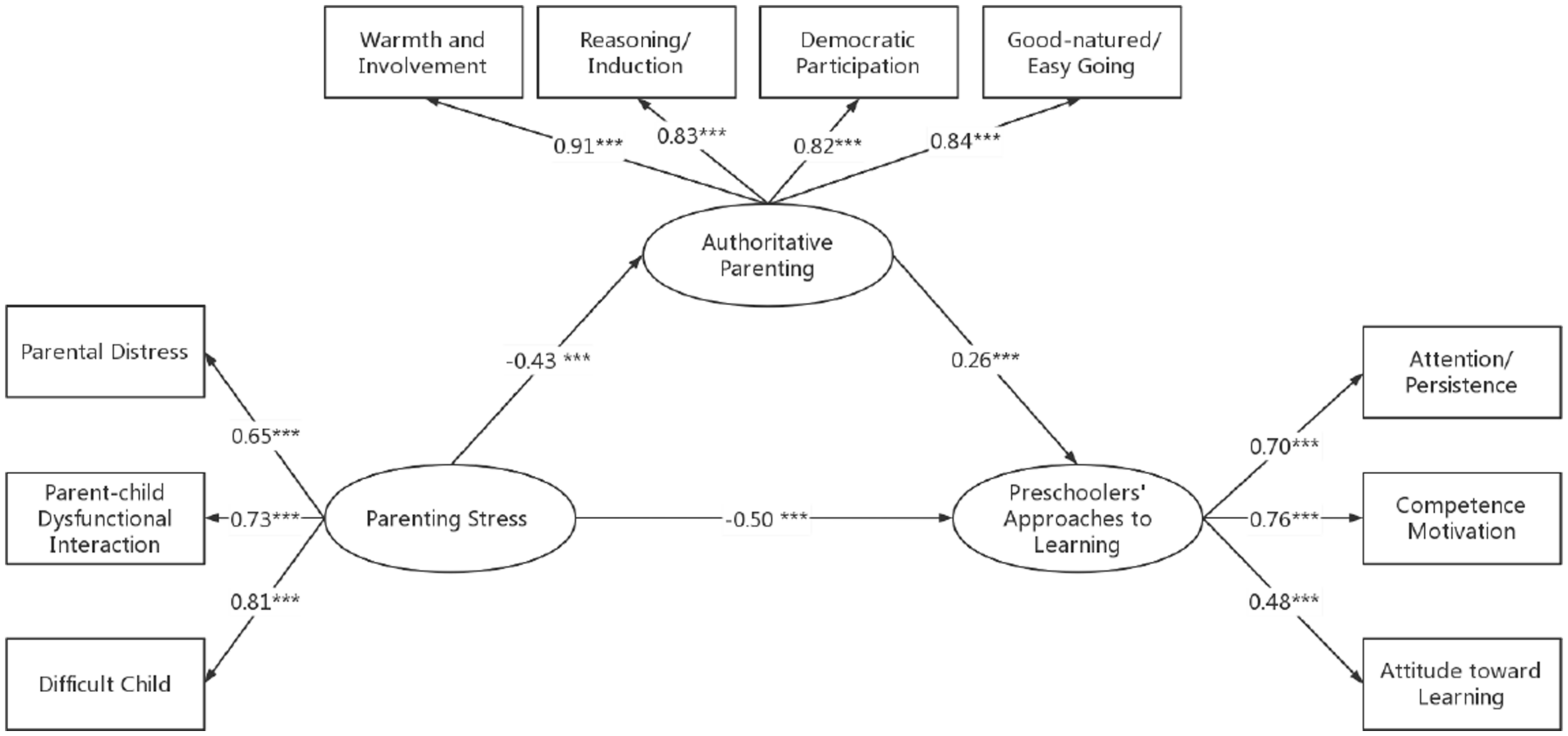
Mediation analysis results. *p < 0.05, **p < 0.01, ***p < 0.001. Gender and age were control variables, which are not shown in Figure, for concise purposes.

Second, the original model also fitted well by including authoritative parenting as a mediating variable, with various fit indices of RMSEA = 0.06, CFI = 0.95, TLI = 0.94, SRMR = 0.05. Parenting stress negatively significantly predicted authoritative parenting (*b* = −0.52, *p* < 0.001) with a 95% confidence interval of [−0.56, −0.48], and authoritative parenting significantly positively predicted preschoolers’ ATL (*b* = 0.10, *p* < 0.001) with the 95% confidence interval was [0.08, 0.11], indicating that a mediating effect holds. The mediating effect size was 0.12, *p* < 0.001, with 95% confidence intervals of [0.10, 0.13], none of the 95% confidence intervals included 0, showing significance, and the mediating effect accounted for 54% of the total effect (0.22). As a result, authoritative parenting mediated the relationship between parenting stress and preschoolers’ ATL. Hypothesis 2 was supported. Figure 2 shows the findings.

### Testing of moderated mediation model

We used multi-group comparison to test whether household residency moderates the relationship between authoritative parenting and preschoolers’ ATL. The result shows that in the tested model, both household residency (natives and migrants) pass the tests of measurement invariance, including Configural Invariance, Metric Invariance, and Scalar Invariance sequentially (Table 3 presents detailed analysis results). Therefore, the model can be applied to different groups. Further, we added pathways between the variables based on the tested model. In order to test whether there are differences between the two groups in the latter half of the mediation model, the pathway between authoritative parenting and preschoolers’ ATL was freely estimated between the two groups. Conversely, the pathway between parenting stress and authoritative parenting as well as the pathway between parenting stress and preschoolers’ ATL were constrained between different groups. Then, the Wald Test is used to compare the difference between the two freely estimated paths. As a result of the Wald Test (Wald test = 23.45, *df* = 1, *p* < 0.001), the results indicated that native and migrant families showed significant differences on these two freely estimated paths.

**Table 3:** Multi-group analyses of different household residency (native and migrant families) on the mediation model.

Model	χ^2^	df	AIC	BIC	CFI	TLI	SRMR	RMSEA	ΔCFI	ΔTLI	ΔRMSEA	Δχ^2^ (Δdf)
Configural	985.35	64	43821.55	44252.30	0.96	0.95	0.04	0.07 [0.07, 0.08]	NA	NA	NA	NA
Metric	1011.24	71	43833.45	44218.51	0.96	0.95	0.05	0.07 [0.07, 0.08]	<0.01	<0.01	<0.01	25.89 (7)***
Scalar	1090.81	78	43899.02	44238.40	0.96	0.95	0.05	0.07 [0.07, 0.08]	<0.01	<0.01	<0.01	79.57 (7)***

***p < 0.001.

In native families, the mediating effect value of authoritative parenting between parenting stress and preschoolers’ ATL was −0.13, 95%CI was [−0.15, −0.11], and the mediating effect was significant, accounting for 21.2%. Among migrant families, the mediating effect was −0.08, 95%CI was [−0.09, −0.06], which was also significant, accounting for 12.8%. The difference in the mediating effect between the two types of families was 0.03, *p* < 0.001. Furthermore, the results show that the mediating effect of native families was significantly greater than that of migrant families. This indicates that household residency moderated authoritative parenting’s mediating effects in the relationship between authoritative parenting and preschoolers’ ATL. Thus, hypothesis 3 was supported.

## Discussion

### The relationship between parenting stress and preschoolers’ approaches to learning

In this study, it was found that parenting stress negatively affected preschoolers’ ATL. The family stress model also emphasizes the impact of family or parental stress on children’s development (Masarik and Conger, 2017). According to the model, parental stress can contribute to a stressful home atmosphere, which can adversely affect the children (Schmiedeberg and Bozoyan, 2021). That is, when children are exposed to such a stressful family environment, they also experience more negative emotions such as feeling neglected, anxious, or depressed. Additionally, these negative feeling can hinder their concentration, flexibility, and creativity, discourage their passion for learning, and further impact their academic performance. Moreover, a parent who is stressed out by parenting tends to behave less affectionately and less patiently toward his or her children and becomes less engaged in their daily routine (Conger and Donnellan, 2007). It is conceivable that low-frequency and low-quality parent–child interactions have a negative impact on children’s performance (Burchinal et al., 2002). Further, when parents experience high levels of parenting stress, children may not receive positive emotional support and responses from parent–child interactions but rather receive more negative feedback (Fonseca et al., 2020). Preschoolers often receive negative feedback or punishment, resulting in a lack of self-confidence, curiosity, exploration motivation, etc. Further, all these factors will inhibit preschoolers’ ATL development. As such, reducing parental stress levels can improve not only the psychological well-being of parents but also the ATL of preschoolers as well (Deater-Deckard, 1998).

### Mediating effect of authoritative parenting

The results showed that the authoritative parenting style mediated the relationship between parenting stress and preschoolers’ ATL. In other words, authoritative parenting buffers the negative impact of parenting stress on preschoolers’ ATL. As such, this pathway fits with the extension of the family stress model (Masarik and Conger, 2017; Lee et al., 2021). The family stress model indicates that parental stress and depression make it difficult for parents to maintain a positive relationship with their children and may lead to a variety of maladaptive parenting practices (Masarik and Conger, 2017). Consequently, children are at a higher risk of displaying problematic behaviors, achieving poor educational outcomes, and experiencing emotional difficulties (Schmiedeberg and Bozoyan, 2021). Firstly, consistent with previous studies, we have found that parenting stress negatively predicts authoritative parenting styles (Cheah et al., 2009). Parents may feel anxious and sullen under high levels of parenting stress, and these negative emotions may reduce their positive responses to their children (Fonseca et al., 2020). Moreover, parents who suffer from high parenting stress may experience self-doubt and low parenting self-efficacy, which leads them to be less involved in their children’s daily lives (Crnic and Low, 2002). Nevertheless, all these behaviors are contrary to authoritative parenting. Secondly, the present study confirmed previous findings by indicating that authoritative parenting was positively related to preschoolers’ ATL (Xia, 2023). Accordingly, authoritative parenting is more conducive to improving preschoolers’ ATL because it displays high levels of warmth, support, and responsiveness. Indeed, parents who adopt authoritative parenting can understand their children’s characteristics better and can help them maximize their strengths (Crnic and Low, 2002). Additionally, authoritative parents are better at communicating with their children, respecting and understanding their children’s feelings, maintaining a positive parent–child relationship, and caring about their children’s school lives. Consequently, children raised in this parenting style will be more independent, confident, and cooperative, as well as more proactive in their learning process, and will exhibit better ATL (Blair, 2002). Thus, parenting stress is not only directly related to preschoolers’ ATL, but it is also indirectly related to preschoolers’ ATL through authoritative parenting practices.

### Moderating effect of household residency

The results of this study showed that household residency can moderate the relationship between authoritative parenting and preschoolers’ ATL. Furthermore, compared with parents in migrant families, native parents’ authoritative parenting showed a stronger positive relationship with preschoolers’ ATL. In our opinion, several factors contributed to this finding. To begin with, economically, the data of the current study showed that migrant preschoolers’ parents in China tend to work in manual labor jobs and have lower household incomes than native families. Moreover, it is well known that capital is vital resource parents provide to their children. Low family income will reduce parents’ investment in their children’s education, including extracurricular books, tutoring, and other education services (Gong and Zhong, 2015). It is imperative to note that this situation is very different from that of native children living in the cities (Gong and Zhong, 2015). Secondly, the result may be explained by the difference in educational background between migrant and native parents. According to the data, it has been found that migrant parents tend to be less educated than parents from native families. However, parents with higher educational levels have a better educational concept and authoritative parenting behavior, which is helpful for the development of children’s ATL (Schady, 2011; Sung and Wickrama, 2018). Highly educated parents generally have effective approaches to supporting their children to learn and measuring their children’s progress positively (Schmiedeberg and Bozoyan, 2021). Further, migrant parents generally work intensively for long hours, which leaves less time for them to be involved in their children’s education (Gong and Zhong, 2015). Additionally, previous research has revealed that parents’ mental health significantly impacts the performance of young children (Herba et al., 2016). Due to the household registration system in China, migrants have difficulty accessing the same urban resources as natives (Knight and Gunatilaka, 2010). Therefore, parents of migrant families may suffer from a sense of deprivation (Xiong et al., 2021). This negative feeling may reduce the effectiveness of their authoritative parenting and further influence children’s ATL (Knight and Gunatilaka, 2010). Furthermore, Yan and colleagues indicated that parents’ perceived social support positively influenced their children’s ATL (Yan et al., 2022). However, migrant parents’ perceived social support is generally lower than native parents, which in turn negatively impacts the child’s ATL (Hashemi et al., 2021). Moreover, the family environment also plays an important role, since migrant families have low-quality housing and a chaotic home environment, which may negatively influence the development of preschoolers’ ATL (Zhao et al., 2023). Thus, native families’ authoritative parenting styles are more conducive to fostering preschoolers’ ATL than migrant families.

## Implication

According to the above, it can be concluded that preschoolers’ ATL is closely associated with parenting stress, authoritative parenting, and household residency. Holly and colleagues suggested that parenting stress may be more influential on both parent and child development than other forms of stress (Holly et al., 2019). High levels of parenting stress can cause parents to become anxious, depressed, vulnerable, and ineffective at parenting, which may adversely affect the development of their children (Neece et al., 2012). Consequently, in order to improve preschoolers’ ATL and help them reach their full potential, reducing parenting stress is crucial. It is possible for parents to alleviate parenting stress through a variety of techniques. These techniques include mindfulness meditation, exercise, reading books related to improving parenting practices, cognitive restructuring, and increasing communication with family members (Neece et al., 2012; Conner and White, 2014). Moreover, since social support is a valuable resource for relieving stress, the government and related institutions can provide parenting guidance or resources to parents (Crnic and Low, 2002). For example, the government can increase financial investment in childcare services and establish a sound childcare system. This will help working parents balance their work and parenting responsibilities. Further, preschools can organize parent support groups or create online communities where parents can connect, share experiences, seek advice and get emotional support from fellow parents. These groups create a sense of belonging and foster a supportive network among parents facing similar challenges.

In addition, authoritative parenting is generally considered to be the most effective parenting style, which is essential for potential early intervention (Crnic and Low, 2002). Children raised by authoritative parenting styles have fewer externalizing problems and better ATL (Pinquart, 2017). Parents can develop authoritative parenting style by receiving support from the government, preschools, or communities. To begin with, the government can establish policies supporting parental leave and appropriate working hours. These policies enable parents to work-life balance and spend quality time with their children, which is crucial for implementing authoritative parenting practices. Secondly, preschools can conduct a variety of parent education lectures where teachers can offer guidance and feedback on parenting approaches for parents. In addition, the community can provide parenting books, articles, and online resources for parents to educate themselves regarding authoritative parenting. Lastly, parents should be familiar with the characteristics of their children and provide them with the appropriate and necessary guidance to foster their development. Importantly, parents should be involved in their children’s daily lives in a warm, supportive, and encouraging way.

Furthermore, this study also provides further evidence that household residency affects the relationship between authoritative parenting style and preschoolers’ ATL. Burchinal et al. (2002) also point out that children’s outcomes were most closely predicted by family characteristics. It has been shown that migrant families are at a disadvantage in accessing urban resources in China (Knight and Gunatilaka, 2010; Xiong et al., 2021). As the number of Chinese migrants continues to increase, we should pay attention to the needs of this special group (Zhao et al., 2023). For example, it may be necessary for the government to reform household registration or adopt more migrant-friendly policies to narrow the resource gap between migrant and native families (Hashemi et al., 2021). Specifically, for migrant preschoolers, the government should improve preschool enrollment policy for migrant children. This can protect migrant preschoolers’ equal rights to preschool education in their urban residence and further foster their ATL development. Moreover, preschools can encourage regular parent-teacher communication to offer more assistance and guidance to migrant parents regarding their families’ education. In addition, communities should assign more professional social workers or conduct various activities to help migrants integrate into the local community. This will be beneficial for ensuring the community integration, education acquisition, and urban adaptation of parents and children from migrant families, thereby promoting the improvement of the ATL of migrant children.

## Limitations and future research

Although examining the effects of parent and home variables, this study is limited by using parent reports to assess preschoolers’ ATL. Even though our assessment is standardized, it may not be as sensitive to ATL as observational measures or individualized criteria assessments. In McDermott’s view, teachers are the most effective and reliable source of observations of children’s behavior in the classroom (McDermott, 1986; Dominguez et al., 2011). As such, teachers’ observations of preschoolers’ ATL based on preschool contexts would be valuable in future research.

Additionally, it is imperative to recognize that although our model assumed a relationship between parenting stress and preschoolers’ ATL, we did not assess this relationship across multiple time periods. Previous studies have indicated that preschoolers’ ATL develops dynamically. Therefore, further research is required to examine whether and how parenting stress influences longitudinal changes in preschoolers’ ATL.

## Conclusion

This study examined the effects of parenting stress on preschoolers’ ATL based on the family stress model. The sample included preschoolers from migrant and native families in China. In addition, a mediating effect of authoritative parenting as well as a moderate effect of household residency was also investigated. Firstly, after controlling for gender and age, parenting stress affected preschoolers’ ATL negatively. Secondly, authoritative parenting mediates the relationship between parenting stress and preschoolers’ ATL. Last but not least, the mediating effects of authoritative parenting were moderated by household residency. Finally, this study contributes to previous research by examining the mechanisms of parenting stress on preschoolers’ ATL. And we also inform efforts to improve ATL among preschoolers in Chinese migrant and native families.

## Data availability statement

The raw data supporting the conclusions of this article will be made available by the authors, without undue reservation.

## Ethics statement

Ethical review and approval for this study was provided by the Guangzhou University School of Education and related research ethics committee. All procedures followed were in accordance with the ethical standards of Institutional Review Board on Human Experimentation of School of Education, Guangzhou University [Guangzhou University, Guangdong Province, China] and with the Helsinki Declaration of 1975, as revised in 2000. Written informed consent to participate in this study was provided by the participants.

## Author contributions

JZ and YF designed the study. JZ collected the data. YF and CL analyzed the data. ZL drafted and revised the main manuscript. JZ and LZ provided valuable ideas and substantial feedback for the study. All authors approved the final version of this manuscript.

## Funding

This work was supported by the Ministry of Education Youth Fund Project for Humanities and Social Sciences Research (18YJC880138).

## Conflict of interest

The authors declare that the research was conducted in the absence of any commercial or financial relationships that could be construed as a potential conflict of interest.

## Publisher’s note

All claims expressed in this article are solely those of the authors and do not necessarily represent those of their affiliated organizations, or those of the publisher, the editors and the reviewers. Any product that may be evaluated in this article, or claim that may be made by its manufacturer, is not guaranteed or endorsed by the publisher.

## References

[ref1] Abidin R. R. (1983). Parenting stress index. Charlottesville, VA, US: Pediatric Psychology Press.

[ref2] Abidin R. R. (1995). Parenting stress index, third edition: professional manual. Odessa, FL, US: Psychological Assessment Resources.

[ref3] Baker B. L. McIntyre L. L. Blacher J. Crnic K. Edelbrock C. Low C. (2003). Pre-school children with and without developmental delay: behaviour problems and parenting stress over time. J. Intellect. Disabil. Res. 47, 217–230. doi: 10.1046/j.1365-2788.2003.00484.x, PMID: 12787154

[ref4] Barglowski K. (2019). Migrants’ class and parenting: the role of cultural capital in migrants’ inequalities in education. J. Ethn. Migr. Stud. 45, 1970–1987. doi: 10.1080/1369183X.2018.1476130

[ref5] Baumrind D. (1971). Current patterns of parental authority. Dev. Psychol. 4, 1–103. doi: 10.1037/h0030372

[ref6] Blair C. (2002). School readiness: integrating cognition and emotion in a neurobiological conceptualization of children’s functioning at school entry. Am. Psychol. 57, 111–127. doi: 10.1037/0003-066X.57.2.111, PMID: 11899554

[ref7] Burchinal M. R. Peisner-Feinberg E. Pianta R. Howes C. (2002). Development of academic skills from preschool through second grade: family and classroom predictors of developmental trajectories. J. Sch. Psychol. 40, 415–436. doi: 10.1016/S0022-4405(02)00107-3

[ref8] Bustamante A. S. White L. J. Greenfield D. B. (2017). Approaches to learning and school readiness in head start: applications to preschool science. Learn. Individ. Differ. 56, 112–118. doi: 10.1016/j.lindif.2016.10.012

[ref9] Chazan-Cohen R. Raikes H. Brooks-Gunn J. Ayoub C. Pan B. A. Kisker E. E.. (2009). Low-income children’s school readiness: parent contributions over the first five years. Early Educ. Dev. 20, 958–977. doi: 10.1080/10409280903362402

[ref10] Cheah C. S. L. Leung C. Y. Y. Tahseen M. Schultz D. (2009). Authoritative parenting among immigrant Chinese mothers of preschoolers. J. Fam. Psychol. 23, 311–320. doi: 10.1037/a0015076, PMID: 19586194 PMC3725299

[ref11] Conger R. D. Donnellan M. B. (2007). An interactionist perspective on the socioeconomic context of human development. Annu. Rev. Psychol. 58, 175–199. doi: 10.1146/annurev.psych.58.110405.085551, PMID: 16903807

[ref12] Conger R. D. Wallace L. E. Sun Y. Simons R. L. McLoyd V. C. Brody G. H. (2002). Economic pressure in African American families: a replication and extension of the family stress model. Dev. Psychol. 38, 179–193. doi: 10.1037/0012-1649.38.2.179, PMID: 11881755

[ref13] Conner C. M. White S. W. (2014). Stress in mothers of children with autism: trait mindfulness as a protective factor. Res. Autism Spectr. Disord. 8, 617–624. doi: 10.1016/j.rasd.2014.02.001

[ref14] Coolahan K. Fantuzzo J. Mendez J. McDermott P. (2000). Preschool peer interactions and readiness to learn: relationships between classroom peer play and learning behaviors and conduct. J. Educ. Psychol. 92, 458–465. doi: 10.1037/0022-0663.92.3.458

[ref15] Crnic K. A. Gaze C. Hoffman C. (2005). Cumulative parenting stress across the preschool period: relations to maternal parenting and child behaviour at age 5. Infant Child Dev. 14, 117–132. doi: 10.1002/icd.384

[ref16] Crnic K. Low C. (2002). “Everyday stresses and parenting” in Handbook of parenting: practical issues in parenting. ed. Bornstein M. H., vol. 5. 2nd ed (Mahwah, NJ, US: Lawrence Erlbaum Associates Publishers), 243–267.

[ref18] Deater-Deckard K. (1998). Parenting stress and child adjustment: some old hypotheses and new questions. Clin. Psychol. Sci. Pract. 5, 314–332. doi: 10.1111/j.1468-2850.1998.tb00152.x

[ref19] Dominguez X. Vitiello V. E. Fuccillo J. M. Greenfield D. B. Bulotsky-Shearer R. J. (2011). The role of context in preschool learning: a multilevel examination of the contribution of context-specific problem behaviors and classroom process quality to low-income children’s approaches to learning. J. Sch. Psychol. 49, 175–195. doi: 10.1016/j.jsp.2010.11.002, PMID: 21530763

[ref20] Fang Y. Luo J. Boele M. Windhorst D. van Grieken A. Raat H. (2022). Parent, child, and situational factors associated with parenting stress: a systematic review. Eur. Child Adolesc. Psychiatry 1–19. doi: 10.1007/s00787-022-02027-1, PMID: 35876894 PMC11211171

[ref21] Fibbi R. Truong J. (2015). Parental involvement and educational success in Kosovar families in Switzerland. Comp. Migr. Stud. 3, 1–17. doi: 10.1186/s40878-015-0010-y

[ref22] Fonseca A. Moreira H. Canavarro M. C. (2020). Uncovering the links between parenting stress and parenting styles: the role of psychological flexibility within parenting and global psychological flexibility. J. Context. Behav. Sci. 18, 59–67. doi: 10.1016/j.jcbs.2020.08.004

[ref23] Gong J. Zhong Z. (2015). The differences in family education between migrant children and urban native children and its influencing factors: based on the analysis of 853 samples in Wuhan (In Chinese). Learn. Pract. 373, 106–114. doi: 10.19624/j.cnki.cn42-1005/c.2015.03.013

[ref24] Gouveia M. J. Carona C. Canavarro M. C. Moreira H. (2016). Self-compassion and dispositional mindfulness are associated with parenting styles and parenting stress: the mediating role of mindful parenting. Mindfulness 7, 700–712. doi: 10.1007/s12671-016-0507-y

[ref25] Harmeyer E. Ispa J. M. Palermo F. Carlo G. (2016). Predicting self-regulation and vocabulary and academic skills at kindergarten entry: the roles of maternal parenting stress and mother-child closeness. Early Child Res. Q. 37, 153–164. doi: 10.1016/j.ecresq.2016.05.001

[ref26] Hashemi N. Marzban M. Sebar B. Harris N. (2021). Perceived discrimination and subjective well-being among middle eastern migrants in Australia: the moderating role of perceived social support. Int. J. Soc. Psychiatry 67, 110–119. doi: 10.1177/0020764020940740, PMID: 32635789

[ref27] Herba C. M. Glover V. Ramchandani P. G. Rondon M. B. (2016). Maternal depression and mental health in early childhood: an examination of underlying mechanisms in low-income and middle-income countries. Lancet Psychiatry 3, 983–992. doi: 10.1016/S2215-0366(16)30148-1, PMID: 27650772

[ref28] Holly L. E. Fenley A. R. Kritikos T. K. Merson R. A. Abidin R. R. Langer D. A. (2019). Evidence-Base update for parenting stress measures in clinical samples. J. Clin. Child Adolesc. Psychol. 48, 685–705. doi: 10.1080/15374416.2019.1639515, PMID: 31393178

[ref29] Horwitz S. M. Irwin J. R. Briggs-Gowan M. J. Heenan J. M. B. Mendoza J. Carter A. S. (2003). Language delay in a community cohort of young children. J. Am. Acad. Child Adolesc. Psychiatry 42, 932–940. doi: 10.1097/01.CHI.0000046889.27264.5E, PMID: 12874495

[ref30] Hosokawa R. Katsura T. (2019). Role of parenting style in children’s behavioral problems through the transition from preschool to elementary school according to gender in Japan. Int. J. Environ. Res. Public Health 16:21. doi: 10.3390/ijerph16010021, PMID: 30577659 PMC6339084

[ref31] Hu B. Y. Teo T. Nie Y. Wu Z. (2017). Classroom quality and Chinese preschool children’s approaches to learning. Learn. Individ. Differ. 54, 51–59. doi: 10.1016/j.lindif.2017.01.007

[ref32] Huang C. Y. Costeines J. Kaufman J. S. Ayala C. (2014). Parenting stress, social support, and depression for ethnic minority adolescent mothers: impact on child development. J. Child Fam. Stud. 23, 255–262. doi: 10.1007/s10826-013-9807-1, PMID: 24653641 PMC3956110

[ref33] Kagan S. L. Moore E. K. Bredekamp S. (1995). Reconsidering children’s early development and learning. Washington, DC, US: Government Printing Office.

[ref35] Kline R. B. (2005). Principles and practice of structural equation modeling, 2nd. New York, NY: Guilford Press.

[ref36] Knight J. Gunatilaka R. (2010). Great expectations? The subjective well-being of rural-urban migrants in China. World Dev. 38, 113–124. doi: 10.1016/j.worlddev.2009.03.002

[ref37] Lee J. Y. Volling B. L. Lee S. J. (2021). Material hardship in families with low income: positive effects of coparenting on fathers’ and mothers’ parenting and children’s prosocial behaviors. Front. Psychol. 12:729654. doi: 10.3389/fpsyg.2021.729654, PMID: 34955959 PMC8696346

[ref38] Li-Grining C. P. Votruba-Drzal E. Maldonado-Carreno C. Haas K. (2010). Children’s early approaches to learning and academic trajectories through fifth grade. Dev. Psychol. 46, 1062–1077. doi: 10.1037/a0020066, PMID: 20822223

[ref39] Luthar S. S. Cicchetti D. Becker B. (2000). The construct of resilience: a critical evaluation and guidelines for future work. Child Dev. 71, 543–562. doi: 10.1111/1467-8624.00164, PMID: 10953923 PMC1885202

[ref40] Marcal K. (2022). Pathways from food and housing insecurity to adolescent behavior problems: the mediating role of parenting stress. J. Youth Adolesc. 51, 614–627. doi: 10.1007/s10964-021-01565-2, PMID: 35091880

[ref41] Martin C. A. Papadopoulos N. Chellew T. Rinehart N. J. Sciberras E. (2019). Associations between parenting stress, parent mental health and child sleep problems for children with ADHD and ASD: systematic review. Res. Dev. Disabil. 93:103463. doi: 10.1016/j.ridd.2019.103463, PMID: 31446370

[ref42] Masarik A. S. Conger R. D. (2017). Stress and child development: a review of the family stress model. Curr. Opin. Psychol. 13, 85–90. doi: 10.1016/j.copsyc.2016.05.008 28813301

[ref44] McClelland M. M. Acock A. C. Piccinin A. Rhea S. A. Stallings M. C. (2013). Relations between preschool attention span-persistence and age 25 educational outcomes. Early Child Res. Q. 28, 314–324. doi: 10.1016/j.ecresq.2012.07.008, PMID: 23543916 PMC3610761

[ref45] McDermott P. A. (1986). “The observation and classification of exceptional child behavior” in Psychological perspectives on childhood exceptionality: a handbook. eds. Brown R. T. Reynolds C. R. (New York, US: Wiley-Interscience), 136–180.

[ref46] McDermott P. A. Leigh N. M. Perry M. A. (2002). Development and validation of the preschool learning behaviors scale. Psychol. Sch. 39, 353–365. doi: 10.1002/pits.10036

[ref47] McDermott P. A. Rikoon S. H. Fantuzzo J. W. (2014). Tracing children’s approaches to learning through head start, kindergarten, and first grade: different pathways to different outcomes. J. Educ. Psychol. 106, 200–213. doi: 10.1037/a0033547

[ref48] McDermott P. A. Rikoon S. H. Fantuzzo J. W. (2016). Transition and protective agency of early childhood learning behaviors as portents of later school attendance and adjustment. J. Sch. Psychol. 54, 59–75. doi: 10.1016/j.jsp.2015.10.003, PMID: 26790703

[ref49] Meade A. W. Johnson E. C. Braddy P. W. (2008). Power and sensitivity of alternative fit indices in tests of measurement invariance. J. Appl. Psychol. 93, 568–592. doi: 10.1037/0021-9010.93.3.568, PMID: 18457487

[ref50] Mistry R. S. Biesanz J. C. Chien N. Howes C. Benner A. D. (2008). Socioeconomic status, parental investments, and the cognitive and behavioral outcomes of low-income children from immigrant and native households. Early Child Res. Q. 23, 193–212. doi: 10.1016/j.ecresq.2008.01.002

[ref51] Moffett L. Weissman A. McCormick M. Weiland C. Hsueh J. Snow C.. (2023). Enrollment in pre-K and children’s social-emotional and executive functioning skills: to what extent are associations sustained across time? J. Educ. Psychol. 115, 460–474. doi: 10.1037/edu0000782

[ref52] Neece C. L. Green S. A. Baker B. L. (2012). Parenting stress and child behavior problems: a transactional relationship across time. Ajidd-Am. J. Intellect. Dev. Disabil. 117, 48–66. doi: 10.1352/1944-7558-117.1.48, PMID: 22264112 PMC4861150

[ref53] Padilla C. M. Ryan R. M. (2020). School readiness among children of Hispanic immigrants and their peers: the role of parental cognitive stimulation and early care and education. Early Child Res. Q. 52, 154–168. doi: 10.1016/j.ecresq.2018.04.008

[ref54] Park H. Walton-Moss B. (2012). Parenting style, parenting stress, and children’s health-related behaviors. J. Dev. Behav. Pediatr. 33, 495–503. doi: 10.1097/DBP.0b013e318258bdb8 22772823

[ref55] Pinquart M. (2017). Associations of parenting dimensions and styles with externalizing problems of children and adolescents: an updated meta-analysis. Dev. Psychol. 53, 873–932. doi: 10.1037/dev0000295, PMID: 28459276

[ref56] Podsakoff P. M. MacKenzie S. B. Lee J.-Y. Podsakoff N. P. (2003). Common method biases in behavioral research: a critical review of the literature and recommended remedies. J. Appl. Psychol. 88, 879–903. doi: 10.1037/0021-9010.88.5.879, PMID: 14516251

[ref57] Razza R. A. Martin A. Brooks-Gunn J. (2015). Are approaches to learning in kindergarten associated with academic and social competence similarly? Child Youth Care Forum 44, 757–776. doi: 10.1007/s10566-015-9307-0, PMID: 26877624 PMC4749025

[ref58] Robinson C. C. Mandleco B. Olsen S. F. Hart C. H. (1995). Authoritative, authoritarian, and permissive parenting practices: development of a new measure. Psychol. Rep. 77, 819–830. doi: 10.2466/pr0.1995.77.3.819

[ref59] Schady N. (2011). Parents’ education, mothers’ vocabulary, and cognitive development in early childhood: longitudinal evidence from Ecuador. Am. J. Public Health 101, 2299–2307. doi: 10.2105/AJPH.2011.300253, PMID: 22021308 PMC3222428

[ref60] Schmiedeberg C. Bozoyan C. (2021). Do economic hardship and pressure really influence parenting? Eur. Sociol. Rev. 37, 287–304. doi: 10.1093/esr/jcaa051

[ref61] Scott-Little C. Kagan S. L. Frelow V. S. (2006). Conceptualization of readiness and the content of early learning standards: the intersection of policy and research? Early child. Res. Q. 21, 153–173. doi: 10.1016/j.ecresq.2006.04.003

[ref62] Shrout P. E. Bolger N. (2002). Mediation in experimental and nonexperimental studies: new procedures and recommendations. Psychol. Methods 7, 422–445. doi: 10.1037/1082-989X.7.4.422, PMID: 12530702

[ref63] Smith-Adcock S. Leite W. Kaya Y. Amatea E. (2019). A model of parenting risk and resilience, social-emotional readiness, and reading achievement in kindergarten children from low-income families model. J. Child Fam. Stud. 28, 2826–2841. doi: 10.1007/s10826-019-01462-0

[ref64] Stephens C. M. Crosby D. A. Yaya-Bryson D. Reid A. (2023). Supporting Spanish-English DLLs in head start: peer language match, instructional language match, and emotional support as predictors of approaches to learning and social skills. Early Child Res. Q. 63, 121–132. doi: 10.1016/j.ecresq.2022.11.005

[ref65] Sung J. Wickrama K. A. S. (2018). Longitudinal relationship between early academic achievement and executive function: mediating role of approaches to learning. Contemp. Educ. Psychol. 54, 171–183. doi: 10.1016/j.cedpsych.2018.06.010

[ref66] Tachibana Y. Fukushima A. Saito H. Yoneyama S. Ushida K. Yoneyama S.. (2012). A new mother-child play activity program to decrease parenting stress and improve child cognitive abilities: a cluster randomized controlled trial. PLoS One 7:e38238. doi: 10.1371/journal.pone.0038238, PMID: 22848340 PMC3407189

[ref67] Tan T. X. Camras L. A. Deng H. Zhang M. Lu Z. (2012). Family stress, parenting styles, and behavioral adjustment in preschool-age adopted Chinese girls. Early Child Res. Q. 27, 128–136. doi: 10.1016/j.ecresq.2011.04.002

[ref68] U.S. Department of Education and National Center for Education Statistics (2002). Early childhood longitudinal study kindergarten class of 1998–99 (ECLS–K), psychometric report for kindergarten through first grade. Available at: https://files.eric.ed.gov/fulltext/ED576531.pdf (Accessed March 26, 2023).

[ref69] Xia X. (2023). Parenting style and Chinese preschool children’s pre-academic skills: a moderated mediation model of approaches to learning and family socioeconomic status. Front. Psychol. 14:1089386. doi: 10.3389/fpsyg.2023.1089386, PMID: 36814671 PMC9940707

[ref70] Xiong M. Xiao L. Ye Y. (2021). Relative deprivation and prosocial tendencies in Chinese migrant children: testing an integrated model of perceived social support and group identity. Front. Psychol. 12:658007. doi: 10.3389/fpsyg.2021.658007, PMID: 34168590 PMC8217643

[ref71] Yan Z. Ren J. Lin W. Wu J. (2022). Parents’ perceived social support and children’s approaches to learning in rural China: a moderated mediation model. Int. J. Environ. Res. Public Health 19:14533. doi: 10.3390/ijerph192114533, PMID: 36361411 PMC9654344

[ref72] Yang X. J. (2013). China’s rapid urbanization. Science 342:310. doi: 10.1126/science.342.6156.310-a 24136949

[ref73] Zhao J. Cui H. Zhou J. Zhang L. (2023). Influence of home chaos on preschool migrant children’s resilience: a moderated mediation model. Front. Psychol. 14:1087710. doi: 10.3389/fpsyg.2023.1087710, PMID: 36925592 PMC10011079

